# EVALUATION OF THE EPIDEMIOLOGY OF EXPOSED FRACTURES BEFORE AND DURING THE COVID-19 PANDEMIC

**DOI:** 10.1590/1413-785220233104e268179

**Published:** 2023-07-31

**Authors:** VINICIUS PAGLIARO FRANCO, GABRIEL MASSARICO GONÇALVES, ORLANDO COPETTI FRAÇÃO, HELOISA YUMI FUJIYA SUNGAILA, LUIZ FERNANDO COCCO, EIFFEL TSUYOSHI DOBASHI

**Affiliations:** 1 Universidade Federal de São Paulo, Escola Paulista de Medicina, Hospital São Paulo, Serviço de Ortopedia e Traumatologia, São Paulo, SP, Brazil.

**Keywords:** Epidemiology, Trauma Centers, Wounds and Injuries, Multiple Trauma, Fractures, Bone, COVID-19, Epidemiologia, Centros de Traumatologia, Ferimentos e Lesões, Traumatismo Múltiplo, Fraturas Ósseas, COVID-19

## Abstract

**Objective:**

To assess the impact of the COVID-19 pandemic on the epidemiology and clinical outcomes of open fractures considering the periods before and during the pandemic.

**Methods:**

An observational and retrospective study, which included patients aged over 18 years, admitted to the Orthopedics and Traumatology Ward of Hospital São Paulo, of the Federal University of São Paulo (UNIFESP). Data was collected in two moments: pre-pandemic (March 1, 2019, to February 29, 2020) and during the pandemic (March 1, 2020, to February 28, 2021).

**Results:**

In total, 183 patients were evaluated with a mean age of 36 years ± 14 years. In the pre-pandemic period, 94 patients underwent surgery, 81 men (85.37%) and 13 women (14.2%), with a mean age of 36 ± 3 years. During the pandemic period, 89 patients were subjected to surgery, 77 men (86.6%) and 12 women (13.4%), with a mean age of 38 ± 3 years.

**Conclusion:**

During the pandemic, open fractures were still more common in men. Regarding hospital indicators, the prevalence of infections in the surgical wound and the length of stay of patients with open fractures increased, however, with little significance. Fractures classified as Gustilo IIIA were the most common, while the most common according to the AO classification were 33, 34, 42, 43, 2R3, and 2R3 + 2U2. The frequency of run overs during the pandemic decreased. However, firearm projectile injuries and falls and occupational injuries increased. ***Level of Evidence III, Retrospective Comparative Study.***

## RESUMO

**Objetivo:**

Avaliar o impacto da COVID-19 na epidemiologia e nos desfechos clínicos das fraturas expostas considerando os períodos antes e durante a pandemia.

**Métodos:**

Estudo observacional e retrospectivo que incluiu pacientes maiores de 18 anos, internados na Enfermaria de Ortopedia e Traumatologia do Hospital São Paulo, da Universidade Federal de São Paulo. Os dados foram coletados em dois momentos - antes (março de 2019 a fevereiro de 2020) e durante a pandemia (março de 2020 a fevereiro de 2021) -, por meio da análise de prontuários eletrônicos de todos os registros hospitalares dessa instituição.

**Resultados:**

No total, foram avaliados 183 pacientes com média de idade de 36 ± 14 anos. No período pré-pandêmico, foram operados 94 pacientes, sendo 81 homens (85,37%) e 13 mulheres (14,2%), com média de idade de 36 ± 3 anos. Já ao longo do período pandêmico, foram operados 89 pacientes, sendo 77 homens (86,6%) e 12 mulheres (13,4%), com média de idade de 38 ± 3 anos.

**Conclusão:**

Durante a pandemia, a ocorrência de fraturas expostas se manteve com maior frequência em indivíduos do sexo masculino. Quanto aos indicadores hospitalares, houve aumento da prevalência de infecções na ferida operatória, assim como do tempo de internação dos pacientes, todavia, com pouca significância. Foram mais frequentes as fraturas classificadas como Gustilo IIIA e, pela classificação AO, as fraturas 33, 34, 42, 43, 2R3 e 2R3 + 2U2. Notamos redução da frequência de atropelamentos durante a pandemia e aumento dos casos de ferimentos por projétil de arma de fogo, quedas e acidentes ocupacionais. **Nível de Evidência III, Estudo Retrospectivo Comparativo.**

## INTRODUCTION

According to a World Health Organization study, COVID-19 has affected over 216 countries, with 194,080,019 confirmed cases and 4,162,304 deaths as of the termination of this study. ^(^ ^1^ The transmission of the SARS-cov-2 disease can easily occur by airways infection and can lead to many clinical situations, ranging from a common cold to more severe respiratory syndromes. ^(^ ^2^

The new scenario set by the COVID-19 pandemic has created enormous pressure on health systems worldwide. This scenario deeply impacted the management of fractures on the locomotor system, which are the main circumstances responsible for admissions to emergency rooms. ^(^ ^3^ Thus, the continuous provision of care in trauma services are a great challenge. ^(^ ^4^ Many orthopedic surgeons have been instructed to postpone or cancel elective surgeries without urgency to delay the transmission of the disease and conserve health resources. ^(^ ^5^

The new standard of living during the pandemic has had a significant impact on epidemiology and prevalence, especially in cases of orthopedic trauma, including fractures. ^(^ ^6^ The increase in social isolation due to the pandemic and the length of stay at home have affected the number of hospital visits and the distribution of patients with fractures who are admitted to the emergency room. ^(^ ^3^

Recent studies such as those by Murphy, Akehurst, and Mutimer^7^ and by Lima et al. ^(^ ^8^ reported a reduction in the number of cases of fractures at the beginning of the pandemic, which probably occurred due to the decrease in patient demand for health services justified by the fear of contamination and the possibility of sequelae from COVID-19 infection. ^(^ ^7^ ^),(^ ^8^ However, we also found studies showing an increase in mortality rates^9^ ^)-(^ ^11^ and complications^12^ ^),(^ ^13^ in patients with fractures treated during the pandemic.

Mortality rates are considerably higher in patients with fractures and seropositive for COVID-19, compared to patients without fractures and seropositive for COVID-19. Regarding the mortality rate in patients with fractures and carriers of COVID-19, a recent systematic review showed an average rate of 34%, and 91.7% mortality rate when considering hip fractures.

Mortality rate was also related to older patients with hip fractures, which is one of the main predictors of death in these patients. ^(^ ^14^ Furthermore, other studies described the consequences of the pandemic in the surgical treatment of different types of fractures, mostly of the hip, ^(^ ^5^ ^),(^ ^14^ lower limbs, ^(^ ^4^ and ankle. ^(^ ^15^ However, only few studies evaluated the impact of the COVID-19 pandemic considering epidemiological data, waiting time for hospitalization, pre- and post-operative follow-up time, treatment options, clinical outcome of patients with open fractures before and during the pandemic. Therefore, information on these aspects is scarce, especially at the national level.

Although many advances in the treatment of open fractures have been achieved over the years, these injuries are still a great challenge, mainly due to the possibility of complications such as infection and non-consolidation, as well as the inherent difficulty of dealing with high-energy injuries with significant impairment of bones and soft tissues. ^(^ ^16^

Furthermore, the COVID-19 pandemic has changed the population’s standards of health care and mobility and has seriously impacted the epidemiology and prevalence of fractures. ^(^ ^8^ Thus, it is essential to understand the impact of the pandemic on the reduction or increase in the prevalence of hospital visits, waiting time for hospitalizations, treatment options, and clinical outcomes of patients with open fractures treated before and during the pandemic, providing information that may improve patient management.

In this way, our research group decided to carry out this study. Therefore, this study aimed to evaluate the impact of the pandemic on patients with fractures considering the epidemiological elements. Moreover, we aimed to analyze the clinical outcome of open fractures in the periods before and during the COVID-19 pandemic.

## METHODS

This study was submitted to evaluation and approved by the Research Ethics Committee of the Federal University of São Paulo (UNIFESP) according to opinion No. 5,609,990 of August 29, 2022, for meeting the guidelines provided for in Resolution 466 of 2012 of the National Health Council regarding the ethical and legal aspects related to studies involving human beings.

This is an observational and retrospective study of patients who were admitted to the Orthopedics and Traumatology Ward of the Hospital São Paulo of UNIFESP. Data were collected before (March 1, 2019, to March 29, 2019) and during the pandemic (March 1, 2020, to February 29, 2021). Patients older than 18 years of both sexes who underwent surgical procedures for fractures were included in the study. Those who had a diagnosis of infection such as pyoarthritis, abscesses, thrombosis, and who did not undergo orthopedic surgeries were excluded from the sample.

Patient data were obtained by consulting the electronic medical record (EMR) of Hospital São Paulo, which has all hospital records (HR) of patients. The information collected included: age, sex, trauma mechanism, anatomical site of fractures classified by the radiographic alphanumeric system AO, preoperative time, length of hospital stay, postoperative time, type of surgery, clinical outcome, cause of fracture, affected side, type of surgery, and functional status. Furthermore, patients’ comorbidities, including smoking, diabetes, hypertension, chronic obstructive pulmonary disease, heart failure, and the need for dialysis were recorded.

Postoperative complications of patients were recorded, including death, coma, use of mechanical ventilator, unplanned intubation, stroke, thromboembolic event (deep vein thrombosis or pulmonary embolism), cardiac arrest, acute myocardial infarction, kidney failure, sepsis, septic shock, return to the operating room, wound dehiscence, deep surgical site infection, and peripheral nerve lesions.

Therefore, our sample included 183 patients with a mean age of 36 years ± 14 years. Before the pandemic, 94 patients were operated, 81 (85.37%) men and 13 (14.2%) women, with a mean age of 36 ± 3 years. During the pandemic, 89 patients were operated, 77 (86.6%) men and 12 (13.4%) women, with a mean age of 38 ± 3 years.

The analyses used the tests of percentage comparisons between the two groups as statistical sufficiency, associating the profile of hospital indicators with the incidence rates and diversity of fractures, as well as the risk factors that involved them. All analyses were performed using STATA program version 16 (2019), with a 5% alpha as the adherence parameter.

Measures of central tendency to determine the parametricity of the values were assessed based on the normal distribution of the mean and its respective standard deviation. Categorical variables were presented by their absolute and relative frequencies to the total sample size.

Statistical significance was considered when p-values were equal to or lower than 0.05, both for the results from the *t*-test, and for those from the proportional difference tests, allowing to verify the existence of significant differences between the parameters before and during the COVID-19 pandemic. P-values higher than 0.05 indicate that the period is insignificant for the measures analyzed.

## RESULTS

Table 1 shows data regarding hospital indicators, length of hospital stay, ICU admission, number of surgical procedures, and Gustilo classification.

**Table 1:** Comparison of hospital indicators in the two periods (before and during the COVID-19 pandemic).

Characteristic	Before the pandemic		During the pandemic		***p* value**
Mean and Confidence Interval			Mean and Confidence Interval		
**Length of hospital stay (days)**	8 (2-14)		9 (1-23)		< 0.05*
**Surgical time**	**AF**	**RF**	**AF**	**RF**	0.06
T 1	48	51%	49	55%	
T 2	46	49%	40	45%	
**ICU**	**AF**	**RF**	**AF**	**RF**	> 0.07
No	92	98%	83	93%	
Yes	2	2.1%	6	7%	
**Death**	**AF**	**RF**	**AF**	**RF**	> 0.07
No	93	99%	87	97%	
Yes	1	1%	2	3%	
**Gustilo classification**	**AF**	**RF**	**AF**	**RF**	
I	4	4%	8	9%	> 0.05
II	13	14%	6	6.7%	> 0.03
III A	74	78%	72	80%	> 0.05
III B	3	3%	1	1.2%	-
III C	-	-	2	1.2%	-

*Statistically significant difference between periods.

AF: Absolute frequency; FR: Relative frequency.

Table 2 shows the results based on the test of difference of proportions, involving the qualitative variables related to the surgical processes in both periods evaluated.

**Table 2:** Proportional comparison of variables involved in surgeries before and during the COVID-19 pandemic.

Trauma mechanism	Before the pandemic		During the pandemic		***p* value**
Running over	AF	RF	AF	RF	
	15	16%	9	10.1%	< 0.03*
**Car** × **guard rail accident**	1	1.2%	-	-	-
**Bicycle Accident**	3	3.7%	4	4.5%	> 0.06
**Motorcycle** × **guard rail accident**	2	2.5%	2	2.2%	-
**Motorcycle** × **automobile accident**	36	38%	37	41.5%	> 0.08
**Motorcycle fall**	7	7.4%	7	7.8%	> 0.09
**Sprains**	2	2.5%	1	1.1%	< 0.04*
**Crushing**	5	5.3%	1	1.1%	< 0.02*
**Assaults**	1	1.2%	-	-	-
**HGI**	2	2%	4	4.5%	< 0.01*
**Animal bite**	-	-	1	1.1%	-
**Fall up to 2 meters**	13	14%	10	11.2%	< 0.05*
**Fall greater than 2 meters**	5	5.3%	3	3.3%	< 0.05*
**Chainsaw**	2	2%	3	3.3%	< 0.04*
**Caved in**	-	-	2	2.2%	-
**Diverse**	-	-	2	2.2%	-

*Statistically significant difference between periods.

AF: Absolute frequency; FR: Relative frequency.

Table 3 shows the diversity of fracture classifications, their frequencies, and proportional comparisons.

**Table 3:** Proportional differences between the incidences and classifications of fractures before and during the COVID-19 pandemic.

AO Fracture Classification	Before the pandemic		During the pandemic		***p* value**
3	AF	RF	AF	RF	-
	1	1.06%	-	-	
11	AF	RF	AF	RF	-
	1	1.06%	-	-	
12	AF	RF	AF	RF	> 0.06
	1	1.06%	1	1.12%	
13	AF	RF	AF	RF	> 0.06
	1	1.06%	1	1.12%	
21	AF	RF	AF	RF	> 0.06
	1	1.06%	1	1.12%	
23	AF	RF	AF	RF	-
	3	3.2%	-	-	
31	AF	RF	AF	RF	-
	1	1.06%	-	-	
32	AF	RF	AF	RF	-
	6	6.3%	-	-	
33	AF	RF	AF	RF	< 0.04*
	5	5.3%	2	2.2%	
34	AF	RF	AF	RF	< 0.03*
	1	1.06%	6	6.7%	
41	AF	RF	AF	RF	-
	1	1.06%	-	-	
42	AF	RF	AF	RF	< 0.02*
	28	29%	14	15.7%	
43	AF	RF	AF	RF	< 0.04*
	9	9.5%	4	4.4%	
44	AF	RF	AF	RF	> 0.06
	6	6.3%	4	4.4%	
54	AF	RF	AF	RF	> 0.05
	2	2%	1	1.12%	
61	AF	RF	AF	RF	-
	-	-	1	1.12%	
70	AF	RF	AF	RF	-
	7	7.4%	-	-	
75	AF	RF	AF	RF	-
	-	-	1	1.12%	
77	AF	RF	AF	RF	< 0.05*
	1	1.06%	3	3.3%	
78	AF	RF	AF	RF	> 0.05
	3	3.2%	4	4.4%	
80	AF	RF	AF	RF	-
	1	1.06%	-	-	
81	AF	RF	AF	RF	-
	1	1.06%	-	-	
82	AF	RF	AF	RF	-
	-	-	2	2.2%	
85	AF	RF	AF	RF	-
	-	-	1	1.12%	
88	AF	RF	AF	RF	-
	-	-	3	3.3%	
89	AF	RF	AF	RF	-
	-	-	1	1.12%	
2R1	AF	RF	AF	RF	-
	1	1.06%	-	-	
2R2	AF	RF	AF	RF	-
	1	1.06%	-	-	
2R3	AF	RF	AF	RF	< 0.03*
	1	1.06%	3	3.3%	
2R3 + 2U2	AF	RF	AF	RF	< 0.02*
	2	2.1%	7	7.8%	
2R2 + 2U22R3	AF	RF	AF	RF	-
	-	-	2	2.2%	
2R3 + 2U3	AF	RF	AF	RF	-
	-	-	2	2.2%	
2R3 + 2U3 + 42	AF	RF	AF	RF	-
	-	-	1	1.12%	
2U1	AF	RF	AF	RF	-
	-	-	1	1.12%	
2U2	AF	RF	AF	RF	-
	-	-	2	2.2%	
2U2 + 78	AF	RF	AF	RF	-
	-	-	2	2.2%	
22U	AF	RF	AF	RF	-
	1	1.06%	-	-	
32 + 41 + 77	AF	RF	AF	RF	-
	-	-	2	2.2%	
32 + 43	AF	RF	AF	RF	-
	-	-	2	2.2%	
33 + 43	AF	RF	AF	RF	-
	1	1.06%	-	-	
41 + 34	AF	RF	AF	RF	-
	1	1.06%	-	-	
42 + 41	AF	RF	AF	RF	-
	1	1.06%	-	-	
42 + 80	AF	RF	AF	RF	-
	1	1.06%	-	-	
42 + 4F2	AF	RF	AF	RF	-
	-	-	9	11%	
42 + 80	AF	RF	AF	RF	-
	1	1.06%	-	-	
42 + 87	AF	RF	AF	RF	-
	-	-	1	1.12%	
42 + 88	AF	RF	AF	RF	-
	-	-	1	1.12%	
4F2	AF	RF	AF	RF	-
	-	-	1	1.12%	
77 + 78	AF	RF	AF	RF	-
	-	-	2	2.2%	
81 + 21	AF	RF	AF	RF	-
	1	1.06%	-	-	
78 + 85 + 87	AF	RF	AF	RF	-
	1	1.06%	-	-	

*Statistically significant difference between periods.

AF: Absolute frequency; FR: Relative frequency.

Table 4 shows the data on the incidence of complications, as well as their proportional differences.

**Table 4:** Proportional differences between the incidence of surgical complications before and during the COVID-19 pandemic.

Complication	Before the pandemic		During the pandemic		***p* value**
**Amputation**	AF	RF	AF	RF	< 0.05*
	1	1.06%	2	2.89%	
**Consolidation delay**	AF	RF	AF	RF	-
	-	-	1	1.1%	
**Fat embolism**	AF	RF	AF	RF	-
	1	1.06%	-	-	
**Infection of OSW**	AF	RF	AF	RF	< 0.04*
	11	11.7%	8	9%	
**Synthesis infection**	AF	RF	AF	RF	-
	4	4.2%	-	-	
**Skin injury**	AF	RF	AF	RF	> 0.10
	1	1.06%	1	1.1%	
**Radial nerve injury**	AF	RF	AF	RF	-
	-	-	1	1.1%	
**Poor reduction**	AF	RF	AF	RF	-
	-	-	1	1.1%	
**Deaths**	AF	RF	AF	RF	-
	-	-	2	2.89%	
**Osteomyelitis**	AF	RF	AF	RF	> 0.10
	2	2.12%	2	2.89%	
**Loss of reduction**	AF	RF	AF	RF	-
	-	-	2	2.89%	
**Loss of Substance**	AF	RF	AF	RF	-
	2	2.12%	-	-	
**Pseudarthrosis**	AF	RF	AF	RF	> 0.06
	2	2.12%	3	3.4%	
**Joint stiffness**	AF	RF	AF	RF	-
	1	1.06%	-	-	
**Surgical revision**	AF	RF	AF	RF	-
	-	-	1	1.1%	
**Loss of limbs**	AF	RF	AF	RF	-
	-	-	1	1.1%	
**Compartment Syndrome**	AF	RF	AF	RF	-
	1	1.06%	-	-	
**No complications**	AF	RF	AF	RF	< 0.06
	60	63%	61	68%	

*Statistically significant difference between periods.

AF: Absolute frequency; FR: Relative frequency; OSW: obstetric surgical wound.

There was a statistically significant difference in the prevalence of men in both periods (p < 0.02). However, we found no difference in age groups between the periods analyzed (p > 0.07).

Regarding hospital indicators, we found a slight statistical difference in the length of stay during the pandemic, as well as in the classifications of Gustilo I, II, and IIIA (Table 1).

In the evaluation of risk factors, we highlight the reduction in the frequency of run overs during the pandemic, the increase in firearm projectile injuries (FPI) and falls and occupational injuries, epidemiological situations involved with measures to restrict activities during the pandemic. Amputations slightly increased, however, at levels very close to statistical insignificance. On the other hand, accidents involving motor vehicles, such as motorcycles and automobiles, did not present statistically significant differences between the periods.

When verifying the incidences and classifications of fractures, not all of them could be compared due to the differences of incidence. Those that occurred simultaneously between the groups and that showed statistically significant differences between the periods were 33, 34, 42, 43, 2R3, and 2R3 + 2U2.

Post-surgical complications were the same before and during the pandemic, except for surgical wound infection. Notably, the absence of post-surgical complications maintained stable percentages and without differences between the two periods evaluated.

## DISCUSSION

Both public and private healthcare services have needed significant adaptations to handle the large influx of patients during the COVID-19 pandemic. There has been a new level of redeployment of surgical and anesthetic teams of all grades to manage the vast workload related to the pandemic, which has greatly impacted service delivery, with the complete suspension of elective orthopedic surgeries and significant changes in the way trauma care is provided. ^(^ ^17^

Considering the risks to which patients and medical staff were exposed in the pandemic, the Department of Orthopedics and Traumatology of the Federal University of São Paulo (UNIFESP) created three protocols in the service that were applied during the pandemic. Thus, the application of these protocols aimed to reduce the risk of infection of patients and healthcare professionals and adapt work, academic, and scientific activities, and orthopedic treatment in the face of the pandemic. ^(^ ^8^

The impact on trauma services was especially related to the need to balance optimal treatment of patients’ injuries with safety and clinical resources. The need to reduce hospitalizations and elective surgeries was reinforced, as well as accepting that conventional surgical decision-making would have to change, with an increase in cases of late reconstruction. However, major orthopedic injuries, including open fractures, had to continue their treatment in emergency cases. Open fractures are complex injuries associated with high rates of complications, including infection and neurological and vascular impairment. ^(^ ^17^

Thus, this study aimed to understand the impact of the pandemic on the reduction or increase in the prevalence of hospital visits, the waiting time for hospitalizations, treatment options, and clinical outcomes of patients with open fractures, comparing the rates obtained in our service before and during the COVID-19 pandemic, to provide information that can improve patient management.

Our results showed a higher prevalence of fractures in men for both periods. This finding is similar to that observed by Tian et al., ^(^ ^18^ who evaluated 111 studies involving 41,429 individuals by a systematic review of the literature, to identify factors involved with the occurrence and resolution of fractures, including those involved with the formation of pseudarthrosis in tibial fractures. According to the authors, many factors significantly influenced the outcomes, including being aged over 60 years old and being a man, occurrence of open fracture, IIIB or IIIC fractures according to the Gustilo classification, among others. ^(^ ^18^ These results are very similar to those identified in this survey.

We observed fewer run overs and more firearm projectile injury and domestic accidents during the COVID-19 pandemic. According to Sephton et al., ^(^ ^19^ the confinement led to a decrease in emergency orthopedic emergency referrals and the number of procedures, as well as resulting in a shift in the injury mechanisms, which became characterized by domestic accidents and some situations of violence, justifying the change in the profile of the trauma mechanism observed in this study.

During the pandemic, the prevalence of surgical wound infections increased. Moreover, the length of stay of patients with open fractures was slightly longer during the pandemic.

Although our findings were significant, we had some limitations since this is single-center study and the sample size is relatively small. However, all patients treated at our tertiary health care institution were compiled for the analyses.

## CONCLUSION

During the pandemic, open fractures maintained their occurrence, which had a higher frequency in men. Regarding hospital indicators, the prevalence of infections in the surgical wound and the length of stay of patients with open fractures increased, however, they were not significant. The fractures classified as Gustilo IIIA were the most prevalent and, according to the AO classification, the most common types were 33, 34, 42, 43, 2R3, and 2R3 + 2U2. Furthermore, during the pandemic, the frequency of run overs decreased, however, FPI and falls and occupational injuries increased.
